# Extensive Mandibular Bone Regeneration Following Iodoform and Lyophilized Bone Grafting in a Patient Initially Indicated for Hemimandibulectomy

**DOI:** 10.1155/crid/5757136

**Published:** 2026-10-28

**Authors:** Renato Zacura, Moema A. Hausen, Marli Gerenutti

**Affiliations:** ^1^ Surgery Department Postgraduation Program of Biomaterials and Regenerative Medicine Faculty of Medical Sciences and Health Pontifical Catholic University of São Paulo São Paulo Brazil pucsp.br

**Keywords:** absorbable bone biomaterials, hemimandibulectomy, iodoform, mandible regeneration, mandibular cysts

## Abstract

Mandibular cysts arise from various etiologies and generally exhibit benign characteristics, with favorable treatment outcomes. Nevertheless, extensive cystic lesions may involve cortical bone, leading to substantial bone loss, reduced mechanical integrity of the mandible, and an increased risk of pathological fractures. In severe cases, hemimandibulectomy may become a clinical option, potentially resulting in functional and aesthetic impairment and requiring complex mandibular reconstruction. Therefore, conservative regenerative strategies aimed at preserving mandibular continuity may provide substantial benefits for patient recovery and quality of life. In this case report, we present a successful approach in which a mandible severely affected by benign cyst‐induced bone loss was preserved and extensively regenerated over a 6‐year follow‐up period. Following preoperative endodontic management with iodoform, the cyst was completely enucleated, and the resulting extensive bone defect was filled with lyophilized bovine bone combined with iodoform and covered with collagen membranes and a titanium mesh. Long‐term radiographic follow‐up demonstrated progressive increases in radiopacity and integration of the treated area, whereas computed tomography at 5.5 years revealed restoration of cortical bone continuity and extensive mandibular remodeling. At 6 years, the treated region exhibited radiopacity similar to that of the contralateral preserved mandibular region. These findings suggest that, in carefully selected extensive benign mandibular lesions, a conservative regenerative approach may preserve mandibular continuity and potentially avoid extensive mandibular resection.

## 1. Introduction

Mandibular cysts arise from multiple etiologies and are generally benign. Because of their characteristics, treatment generally presents a favorable prognosis. However, extensive cystic lesions may involve the cortical bone of the mandible, leading to substantial bone loss and reduced mechanical strength, which may predispose the mandible to pathological fractures [1, 2].

The main types of cystic and cyst‐like lesions can be identified according to their clinical incidence, including dentigerous cysts, keratocystic odontogenic tumors, radicular cysts, and ameloblastomas, with the highest prevalence associated with radicular cysts [3]. Despite numerous clinical approaches aimed at preventing extensive bone resection, which may result in permanent functional loss and disfigurement, extensive mandibular involvement may ultimately require segmental resection or hemimandibulectomy. This approach has been reported even for benign odontogenic lesions when their size and anatomical extension substantially compromise the mandibular structure [4]. On the other hand, efforts to preserve mandibular bone are based on conservative surgical management, including cyst enucleation. The main clinical approaches may include marsupialization and decompression, tooth extraction or traction, enucleation, curettage, and bone resection, depending on the characteristics and extent of the lesion [5].

When hemimandibulectomy turns out to be the final clinical approach, diverse techniques and materials may be indicated for hemimandibular reconstruction. However, extensive bone resorption commonly compromises the prosthetic rehabilitation of these patients [6]. Although there are isolated cases in which spontaneous bone regeneration may occur [7, 8], this is an exceedingly rare phenomenon. Therefore, when the underlying cause of the lesion is not malignant, there are legitimate clinical efforts aimed at cyst removal and restoration of the mandibular bone.

The rationale for the use of iodoform in endodontic treatment is related to its antimicrobial properties. Repeated administration of iodoform through the root canals may contribute to reducing the local bacterial load and promoting a favorable environment for healing prior to surgical intervention [9, 10]. This approach aims to prepare the affected area for subsequent procedures, considering the complex nature of cystic lesions and their effects on the mandibular bone structure.

In this case study, we present a clinical case involving the preservation of a severely compromised mandible affected by substantial bone loss. Because of the extensive bone involvement and fragility observed at the initial evaluation, hemimandibulectomy was initially considered as a treatment option. However, following patient consent, a conservative treatment approach was proposed in an attempt to avoid extensive mandibular resection. After surgical procedures including enucleation, curettage, and preparation of the bone defect, we describe a potentially lower cost method aimed at promoting extensive mandibular bone healing. This method involved the application of iodoform combined with lyophilized bovine bone as graft material. Iodoform was used as an adjunct for local infection prophylaxis, whereas bovine bone provided an osteoconductive scaffold to support bone regeneration. During a prolonged 6‐year follow‐up, the treatment resulted in preservation of mandibular continuity and progressive bone regeneration, supporting the potential application of this conservative approach in selected cases of extensive benign mandibular lesions.

## 2. Clinical History and Diagnosis

A 28‐year‐old male patient initially presented in 2011, when a panoramic x‐ray revealed a cystic lesion, as shown in Figure 1A. At that time, because of the asymptomatic condition, the patient discontinued medical follow‐up. However, in February 2015, after experiencing pain in the right posterior mandibular region, the patient was examined by the first author of this study. Following clinical examination, localized edema associated with facial asymmetry on the right mandibular side was identified, leading to a new panoramic x‐ray examination. This x‐ray revealed an extensive lesion involving the posterior region of the mandibular body and the ascending ramus (Figure 1B). Subsequently, computed tomography (CT) was performed to assess the extent of the lesion and cortical involvement (Figures 2 and 3). Because of the extensive lesion and substantial mandibular involvement, the patient was initially referred for hemimandibulectomy. Following fine‐needle aspiration (FNA), anatomopathological examination revealed a nonmalignant lesion characterized by connective tissue with an inflammatory profile. Despite its benign characteristics, the extensive involvement of the mandible was the main factor supporting the initial indication for hemimandibulectomy.

**Figure 1:**
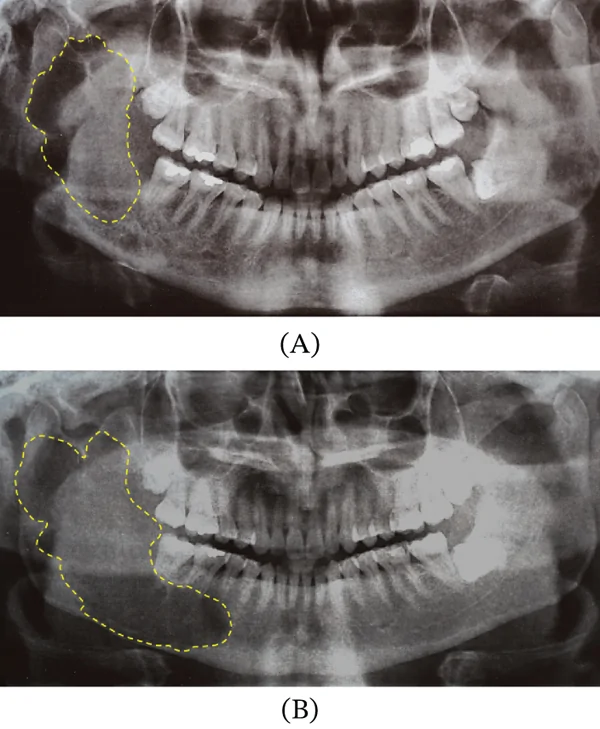
(A) Panoramic x‐ray showing the mandibular pathological lesion identified in May 2011. (B) After approximately 4 years, in February 2015, the lesion had increased in size, resulting in extensive involvement of the mandibular body. Yellow‐dashed lines delineate the lesion, which is characterized by low radiopacity and a homogeneous appearance.

**Figure 2:**
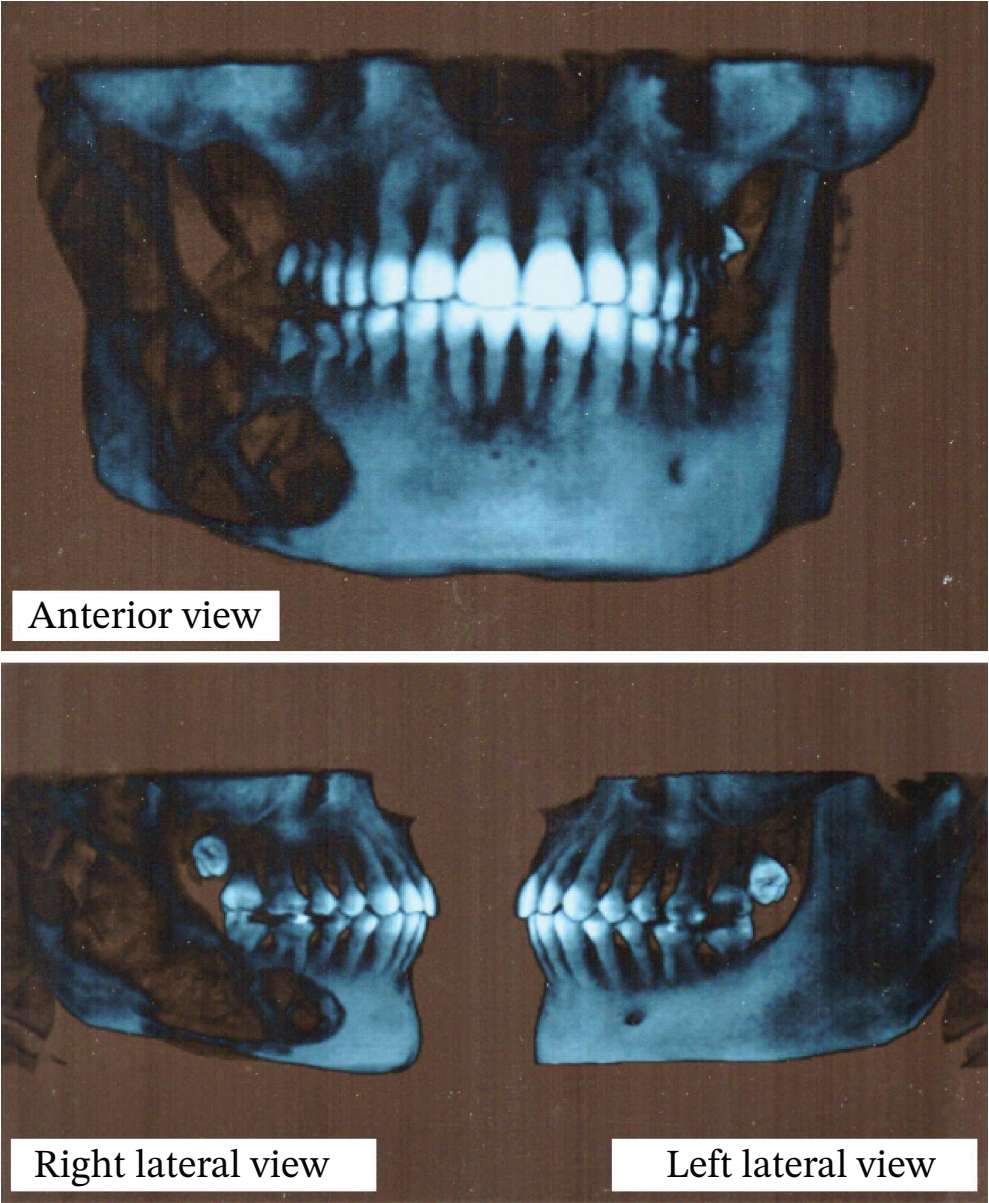
Mandibular CT demonstrates extensive cortical bone loss involving the mandibular body (March 2015).

**Figure 3:**
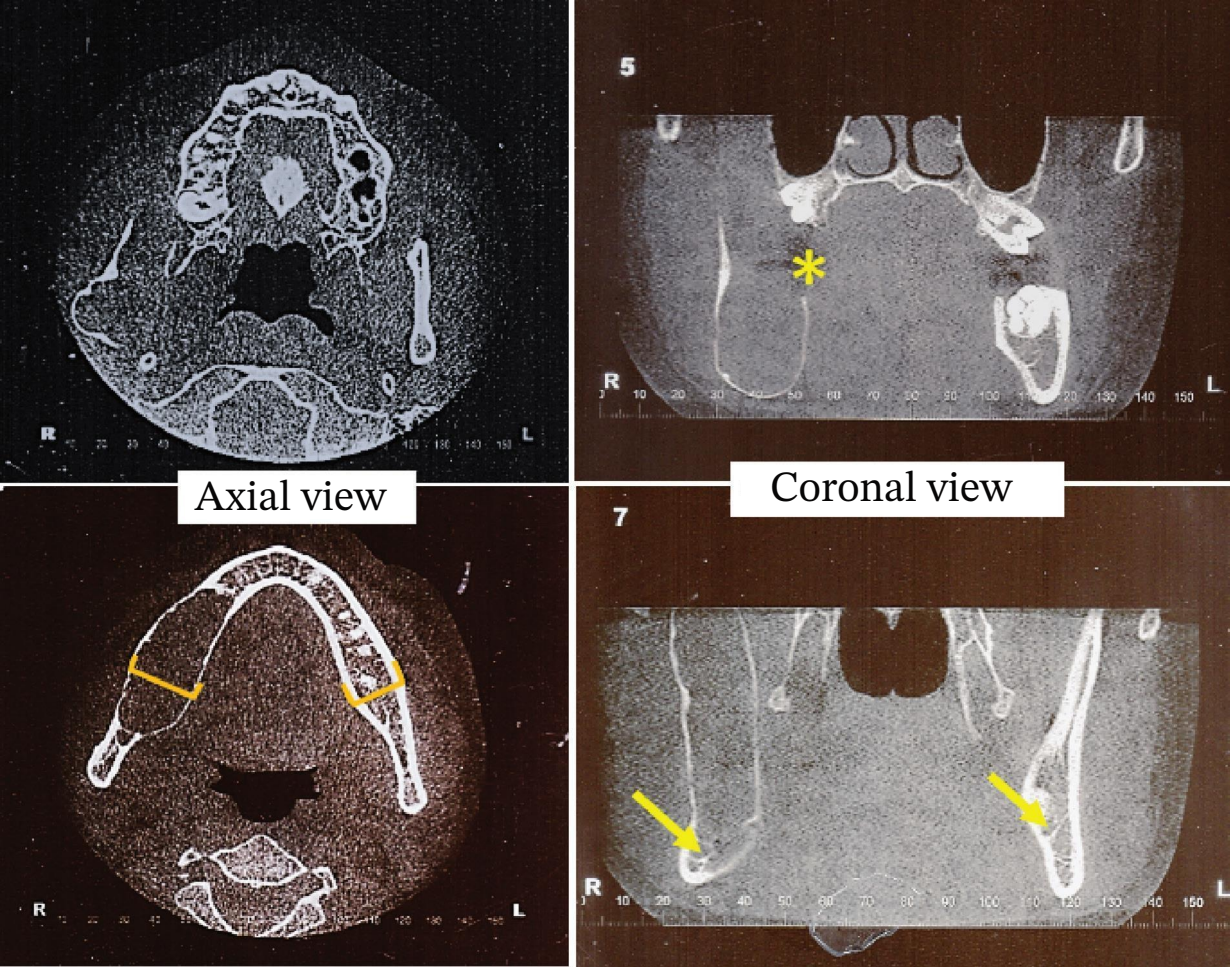
Mandibular CT showing the bone lesion involving the medial aspect of the right ascending ramus. The asterisk (∗) indicates loss of cortical bone; brackets indicate the marked difference in width between the right and left cortical bone; and arrows indicate compression and inferior displacement of the right mandibular canal compared with the left mandibular canal (March 2015).

## 3. Preoperative Evaluation

The histopathological evaluation revealed nonspecific odontogenic cyst. Given the severity and extent of the lesion and the involvement of Teeth 46 and 47, endodontic treatment was considered necessary before surgical intervention to minimize the risk of infection. Prior to definitive endodontic treatment, the patient underwent biweekly sessions over a 16‐week period, during which iodoform was administered through the root canals. This approach aimed to promote antiseptic activity and reduce the local microbial burden as a prophylactic measure. Endodontic treatment was subsequently completed through a series of interventions. Preoperative iodoform administration was intended to reduce microbial contamination and chronic inflammatory activity, thereby providing a more favorable biological environment for subsequent surgical enucleation and bone regeneration. Following the successful completion of this phase, the patient proceeded to surgical treatment.

## 4. Surgical Procedure

In February 2016, the patient underwent surgery at the surgical center of Samaritano Hospital, Sorocaba, São Paulo, Brazil, under total intravenous anesthesia (TIVA). The first incision extended from the ascending ramus to the anterior mandibular region, followed by a second releasing incision extending to the buccal mucosa. After periosteal elevation and dissection of the associated muscles, surgical access to the lesion site was achieved. Following adequate exposure of the lesion, complete cyst enucleation was performed. Macroscopically, the excised lesion consisted of an extensive fibrous tissue capsule. Bone curettage was subsequently performed, allowing complete visualization of the internal aspect of the affected cortical bone and preparation of the resulting bone defect for grafting. The graft consisted of lyophilized bovine bone (Geistlich Bio‐Oss) combined with iodoform (K‐dent Dental Systems Inc.) at a 1:10 ratio (iodoform:bovine bone). The graft was placed within the bone defect and covered with an absorbable collagen membrane (R.T.R., Septodont Inc., United States). A titanium mesh (Neodent Inc.) was then positioned and fixed with titanium screws. A second absorbable collagen membrane was placed over the titanium mesh to prevent direct contact between the metallic material and the periosteum. The surgical site was sutured, and the patient underwent clinical follow‐up at 1, 10, and 45 days postoperatively to monitor healing and recovery.

## 5. Postoperative Evaluation

The excised soft tissue collected during the surgical procedure confirmed the benign nature of the lesion previously identified by the preoperative biopsy. The treated area was evaluated by panoramic x‐rays during a 6‐year follow‐up period, as shown in Figure 4. At 30 days postsurgery, the grafted area remained less radiopaque than the surrounding healthy bone, allowing the boundaries of the original cystic lesion to be identified. After 2 years, an increase in radiopacity was observed in the grafted region, indicating progressive bone formation. After 3 years, advanced bone regeneration and radiographic integration with the surrounding bone were observed. At 6 years, the treated area presented radiopacity similar to that of the contralateral preserved mandibular region. Approximately 1 month before the 6‐year radiographic evaluation, half of the titanium mesh and two screws were removed due to local exposure of the mesh, which was causing injury to the buccal mucosa. Only the exposed portion of the mesh was removed in order to avoid a more extensive surgical intervention, whereas the remaining portion was maintained in place.

**Figure 4:**
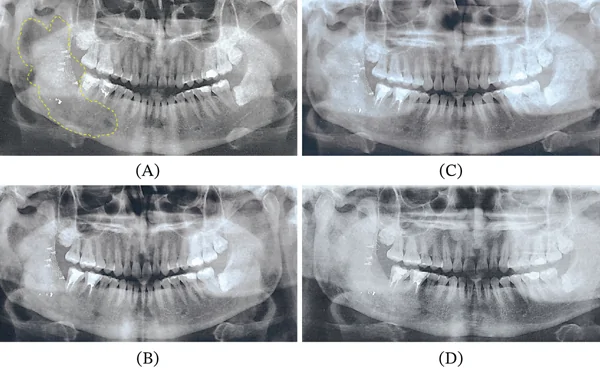
Postoperative panoramic radiographic follow‐up. (A) At 30 days postsurgery, the filled area is still less radiopaque revealing the previous cystic area (yellow‐dashed area). (B) Two years later, increased radiopacity is observed in the filled region. (C) After 3 years, advanced bone regeneration and radiographic integration are observed. (D) After 6 years, the radiopacity of the treated area is similar to that of the contralateral preserved mandibular region. One month before this radiographic examination, approximately half of the titanium mesh and two screws were removed due to the local exposure of the mesh in the oral cavity.

Long‐term tomographic evaluation further confirmed complete cortical bone regeneration and extensive bone remodeling, as evidenced by CT imaging performed 5.5 years after surgery (Figure 5).

**Figure 5:**
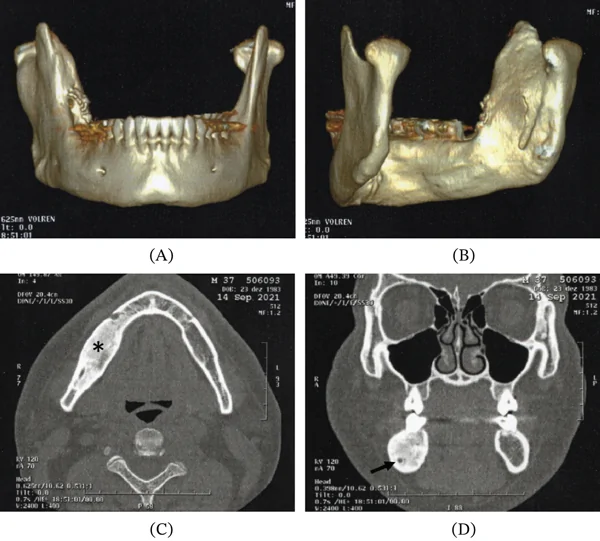
At 5.5 years after surgical grafting, CT imaging revealed the full recovery of the cortical bone in both (A) vestibular and (B) lingual mandible region, as shown in the 3D reconstructions. The (C) axial section reveals bone remodeling (∗), whereas the (D) coronal section depicts (arrow) an ectopic placement of the mandibular canal due to displacement caused by the cyst expansion.

## 6. Discussion

Hemimandibulectomy may be indicated when the mandibular body is extensively compromised by different pathological conditions, including benign or malignant tumors, extensive odontogenic cysts, and osteomyelitis, as previously described by several authors [4, 11, 12]. In some cases, the pathological process involves not only the mandibular body but also the ascending ramus. In such circumstances, segmental mandibular resection or hemimandibulectomy may become necessary. Nevertheless, successful conservative approaches aimed at preserving or regenerating the mandible in selected extensive benign lesions have also been reported [10].

The therapeutic regenerative approach, however, must consider the biological nature of the lesion. In malignant tumors with extensive mandibular involvement, adequate oncological resection and disease control remain the primary objectives, and preservation of compromised mandibular tissue may not be appropriate. Conversely, in selected benign lesions, particularly when oncological margins are not required, conservative strategies aimed at preserving mandibular continuity should be considered whenever clinically feasible. Such approaches may require prolonged treatment, patient compliance, multiple surgical interventions, long‐term follow‐up, and access to appropriate regenerative materials. Despite these limitations, preservation of mandibular continuity may provide substantial functional and aesthetic benefits. Mandibular resection can result in facial asymmetry and impairment of mastication, speech, swallowing, and occlusion, with consequent effects on the patient′s quality of life [13, 14].

In the present case, approximately two‐thirds of the right mandibular body was affected, with complete involvement of the ascending ramus. Extensive benign mandibular lesions with a comparable degree of involvement may require segmental resection or hemimandibulectomy. Farzad [4], for example, reported a large keratocystic odontogenic tumor affecting the right hemimandible and extending to the condylar neck, for which extensive mandibular resection and subsequent reconstruction were required. Given the benign nature of the cyst identified before surgery by histopathological examination in the present case, a conservative strategy was selected to preserve and regenerate the affected mandible. This approach involved prolonged preoperative management, cyst enucleation, bone grafting, and long‐term follow‐up extending to 6 years.

Previous studies have reported conservative management of radicular and other odontogenic cysts using intracanal iodoform‐based formulations, with the aim of treating infection and avoiding tooth extraction [15]. Marsupialization and decompression have also been employed to reduce the dimensions of extensive odontogenic cystic lesions before definitive surgical treatment. Iodoform, frequently associated with other compounds such as calcium hydroxide, has been used as an antiseptic component in the conservative treatment of periapical lesions [9, 16]. Commercially available formulations, such as Endoflas FS, also contain iodoform in combination with other radiopaque and antimicrobial components for endodontic applications.

Hongzhi et al. [17] reported the treatment of 27 patients presenting keratocystic odontogenic tumors with extensive mandibular involvement. Following marsupialization and cyst enucleation, iodoform‐impregnated gauze was used as part of a low‐cost therapeutic approach that was effective in treating infection and preventing recurrence. However, after 1 year of follow‐up, bone recovery remained limited, with incomplete restoration of the cortical bone. In the present case, iodoform was combined with lyophilized bovine bone to fill the extensive defect after cyst enucleation, providing both local antiseptic support and an osteoconductive scaffold for bone regeneration.

The healing mechanism observed in this case may therefore be interpreted as a multifactorial regenerative process rather than as an effect attributable to a single component of the treatment. Complete cyst enucleation and curettage removed the pathological tissue and exposed the residual viable bone surfaces, whereas the bovine bone graft provided an osteoconductive scaffold that may have supported cellular migration, vascular ingrowth, and progressive bone deposition from the surrounding host bone. The collagen membranes and titanium mesh may also have contributed by protecting and maintaining the regenerative space, limiting soft‐tissue invasion and preserving the architecture of the extensive defect during healing. Within this protected osteoconductive environment, progressive host bone formation and subsequent remodeling may explain the gradual radiographic integration and restoration of cortical continuity observed during long‐term follow‐up. Because this is a single case involving multiple simultaneous interventions, the individual contribution of each component to the regenerative process cannot be determined.

The longitudinal radiographic findings demonstrated progressive changes in the treated area. At 30 days postoperatively, the original defect remained clearly distinguishable and showed lower radiopacity than the surrounding mandibular bone. After 2 years, increased radiopacity within the grafted area indicated progressive mineralization and bone formation. At 3 years, the boundaries of the original lesion became substantially less evident, indicating advanced integration of the regenerated area with the surrounding bone. At 6 years, the treated region exhibited radiopacity similar to that of the contralateral preserved mandibular region. Complementary CT evaluation at 5.5 years further demonstrated restoration of cortical bone continuity and extensive remodeling of the reconstructed mandibular region.

These long‐term findings are particularly relevant considering the initial extent of mandibular involvement. Although conclusions regarding the efficacy of this therapeutic approach cannot be generalized from a single case, preservation of mandibular continuity associated with progressive bone regeneration over a 6‐year follow‐up suggests that conservative regenerative strategies may represent an alternative in carefully selected patients with extensive benign mandibular lesions. The present case therefore highlights the potential of combining appropriate lesion management, infection prophylaxis, an osteoconductive graft, and long‐term follow‐up to preserve mandibular anatomy and avoid the functional and aesthetic consequences associated with extensive mandibular resection.

## 7. Conclusion

After the surgical procedure previously described, over a follow‐up period of up to 6 years postsurgery, the patient presented extensive bone regeneration and restoration of mandibular continuity, as demonstrated by tomographic and radiographic evaluations. Iodoform was used as an adjunct for local infection prophylaxis, whereas lyophilized bovine bone grafting provided an osteoinductive scaffold within the regenerative site. The observed bone regeneration likely resulted from the combined effects of lesion removal, preservation of viable host bone, graft osteoconduction, maintenance of the regenerative space, and physiological bone healing and remodeling. The favorable outcome observed in this report supports this conservative approach as a potential alternative to hemimandibulectomy in specific benign conditions, potentially avoiding extensive mandibular resection while promoting bone preservation and regeneration. Such strategies may contribute to improved functional outcomes and patient quality of life.

## Funding

No funding was received for this manuscript.

## Ethics Statement

The ethical Approval Number CAAE: 65640322.6.0000.5373 was obtained through the CEP/CONEP/Brazil Platform. The patient provided written informed consent for the publication of this case report and the accompanying images.

## Conflicts of Interest

The authors declare no conflicts of interest.

## Data Availability

The data that support the findings of this study are available from the corresponding author upon reasonable request.
